# Zika virus baculovirus-expressed envelope protein elicited humoral and cellular immunity in immunocompetent mice

**DOI:** 10.1038/s41598-021-04713-7

**Published:** 2022-01-13

**Authors:** Minna Shin, Kiju Kim, Hyo-Ji Lee, Rangyeon Lee, Yu-Jin Jung, Jeongho Park, Tae-Wook Hahn

**Affiliations:** 1 College of Veterinary Medicine and Institute of Veterinary Science, Kangwon National University, Chuncheon, 24341 Republic of Korea; 2 College of Biological Sciences, Kangwon National University, Chuncheon, 24341 Republic of Korea

**Keywords:** Infectious diseases, Disease prevention

## Abstract

Zika virus (ZIKV) is a mosquito-borne virus that has a high risk of inducing Guillain–Barré syndrome and microcephaly in newborns. Because vaccination is considered the most effective strategy against ZIKV infection, we designed a recombinant vaccine utilizing the baculovirus expression system with two strains of ZIKV envelope protein (MR766, Env_M; ZBRX6, Env_Z). Animals inoculated with Env_M and Env_Z produced ZIKV-specific antibodies and secreted effector cytokines such as interferon-γ, tumor necrosis factor-α, and interleukin-12. Moreover, the progeny of immunized females had detectable maternal antibodies that protected them against two ZIKV strains (MR766 and PRVABC59) and a Dengue virus strain. We propose that the baculovirus expression system ZIKV envelope protein recombinant provides a safe and effective vaccine strategy.

## Introduction

Zika virus (ZIKV) is a mosquito-borne flavivirus classified within the Flaviviridae family that was first isolated in 1947 from rhesus macaques in the Zika Forest, Uganda. Infection with the virus results in Guillain-Barré syndrome in adults and neonatal microcephaly^1–3^. The possibility of global spread is growing because ZIKV has been detected in 87 countries and its transmission vector, *Aedes aegypti* is found in 61 countries^4^. Despite the risk of a pandemic, no authorized ZIKV vaccines are yet available and only a few symptomatic therapies with acetaminophen, antihistamine drugs, and deltamethrin have been used^5,6^.

ZIKV is a single-stranded positive-sense RNA virus (~ 11 kb) composed of a 5′ untranslated region (UTR) (~ 107 nt), an open reading frame (ORF) (10.2 kb), and a 3′ UTR (~ 428 nt). The ORF encodes three structural proteins (capsid, premembrane/membrane (prM), envelope (E)) and seven nonstructural proteins (NS1, NS2A, NS2B, NS3, NS4A, NS4B, and NS5)^7,8^. The capsid protein binds to viral RNA and shapes the nucleocapsid, prM prevents premature fusion with the host-cell membrane, and the E protein mediates receptor binding, cellular attachment, entry, and fusion. The nonstructural proteins inhibit the host antiviral response by regulation of viral transcription, replication, and assembly^9,10^. The role of the ZIKV E protein in vaccine development is critical because it is a major target of neutralizing antibodies and essential for viral invasion, and it elicits protective T cell and antibody responses^11–14^.

Over several decades, vaccine strategies have included purified-inactivated, live-attenuated, DNA, mRNA, and recombinant vaccines^15^. In this study, we developed a ZIKV recombinant subunit vaccine (Env_M, Env_Z) utilizing the E proteins from an epidemic Brazilian strain (ZBRX6) and the original strain (MR766). Animals immunized with the recombinant subunit vaccine demonstrated induced humoral and cellular immunity against these two ZIKV strains. Furthermore, the protective immunity was transmitted to their progeny and they were even protected from a strain of Dengue virus (DENV). To summarize, we designed a ZIKV recombinant subunit vaccine incorporating the baculovirus expression system that is potentially applicable to humans because of its higher level (~ 1.5 times) of recombinant protein production than bacterial systems and its ability to incorporate mammal-like posttranslational modifications^16^.

## Results

### Expression of recombinant ZIKV E protein and induced humoral immunity to ZIKV

**Figure 1:**
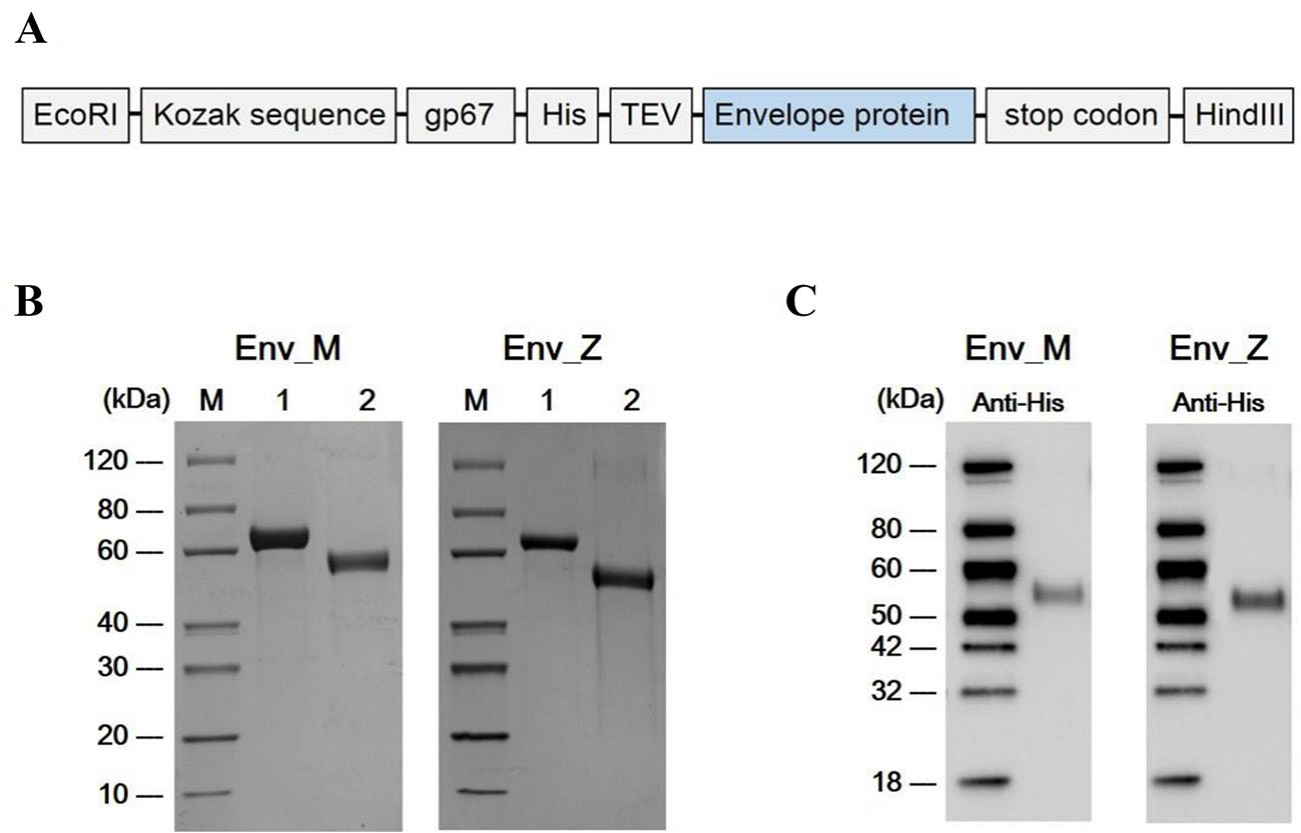
Construction of recombinant ZIKV envelope protein from African (Env M, MR766) Brazilian strain (Env Z, ZBRX6) with baculovirus expression system. (**A**) Schematic of constructs expressing Env_M and Env_Z. (**B**,**C**) Purified recombinant ZIKV Env_M and Env_Z proteins were confirmed by SDS-PAGE (B) and Western Blot (**C**). (**B**) Lane M: Protein Marker (GenScript, Cat. No. M00516). Lane 1: BSA (2.00 μg). Lane 2: Target protein (Env_M or Env_Z 2.00 μg). (**C**) The primary antibodies used anti-His antibody (Mouse-anti-His mAb; GenScript, Cat.No. A00186).

**Figure 2:**
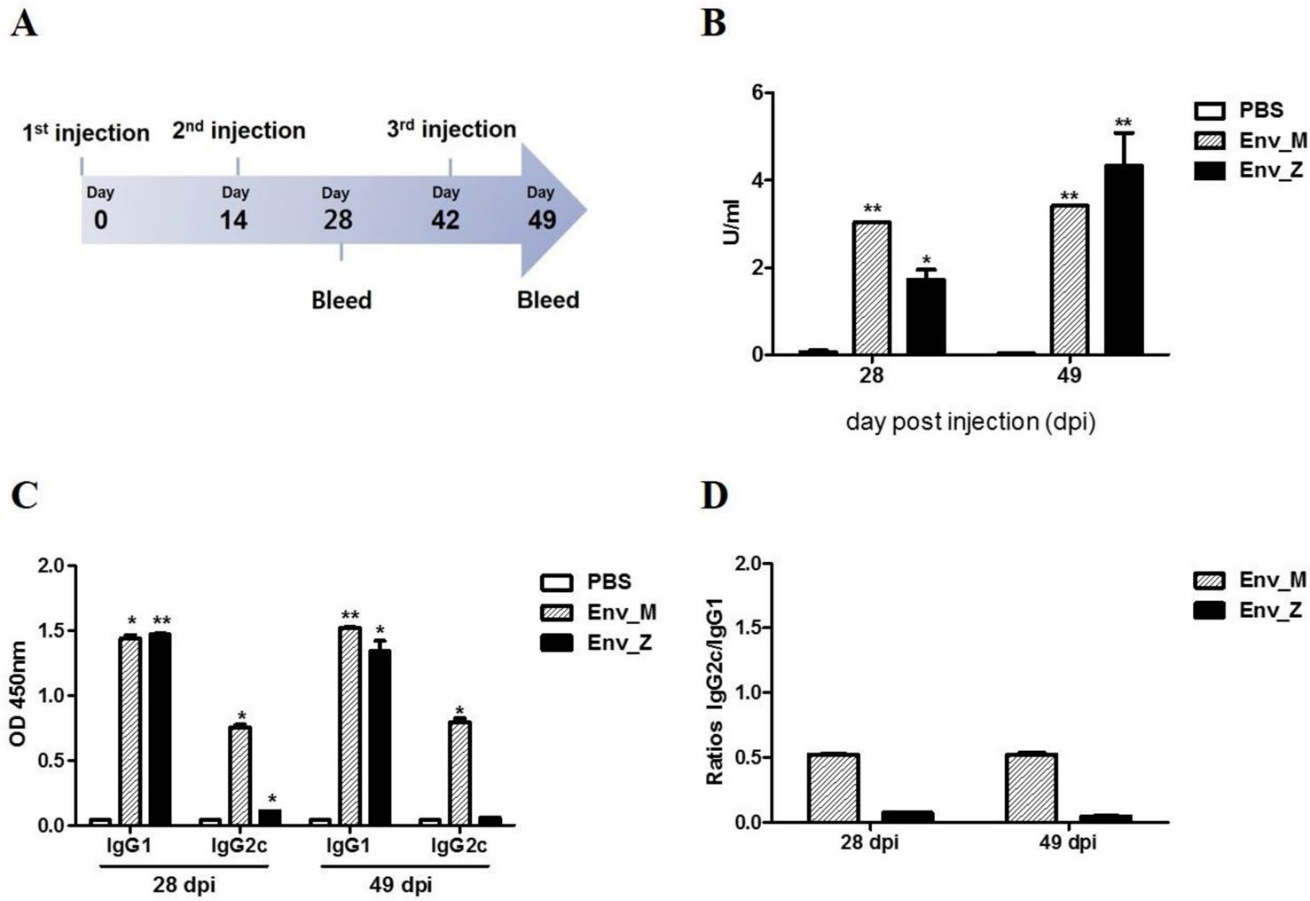
Recombinant ZIKV envelope vaccine elicited Env_M- and Env_Z -specific IgG induction. (**A**) Experimental strategies. C57BL/6 mice (n = 5) were immunized with ZIKV Env_M, Env_Z or PBS at weeks 0, 2 and 6. To measured humoral immune response, blood samples were collected at weeks 0, 4 and 7. (**B**) Serum samples were diluted 1:50,000 and measured for OD 450 values by using ZIKV Envelope protein coated ELISA kit (Alpha diagnostic international). Calculations of ZIKV ENV specific IgG antibody activity units. (**C**) Serum samples were diluted 1:100 measured for OD 450 values by using indirect ELISA. (**D**) Ratios of IgG2c to IgG1 antibodies in Env_M, Env_Z or PBS immunized mice. The data were represented the mean ± SD of five mice per group. Each sample was assayed in duplicates. Statistical analysis was performed comparing immunized group with PBS injected animals and represented *p < 0.05, **p < 0.01, ***p < 0.001.

As a major target of neutralizing antibodies, the ZIKV E protein stimulates effector CD4 + and CD8 + T cell responses and B cell activation^11–14,17^. In this study, we inserted a DNA fragment encoding the E proteins of MR766 (African, Uganda strain) or ZBRX6 (Asian, Brazilian strain) into the pFastBAC1 bacmid under the control of the baculovirus Pph to generate the bacmid pFB-E (Fig. 1A). After purifying the protein with nickel-nitrilotriacetic acid Sepharose beads, expression of the corresponding protein (55 kDa) was confirmed using SDS-PAGE and Western blotting (Fig. 1B,C), and these recombinant vaccines were named Env_M (MR766) and Env_Z (ZBRX6). Humoral immunity is critical for viral clearance and induces protective immune responses via specialized mechanisms such as Fc-mediated effects (antibody-dependent cell-mediated cytotoxicity and complement-dependent cytotoxicity)^18,19^. To evaluate the protective response, we measured E protein-specific IgG using ELISA and found that the antibody titer was significantly increased four weeks after immunization with Env_M and Env_Z (Table 1, Fig. 2A). For example, Env_M inoculation induced the level of antigen-specific antibody by 4,600-folds during the first four weeks and an additional increase (~ 20%) was observed during the following three weeks. Env_Z immunization boosted the antibody level by 2,600-folds by four weeks after immunization followed by an additional increase (2.5-folds) over the next three weeks (Fig. 2B). This result indicates that both Env_M and Env_Z are potent inducers of E protein-specific antibody.

**Table 1:** Zika virus vaccine candidates.

Groups	Vaccine (10 μg/mouse)	Adjuvant (μg/mouse)	No. of mice	Immunization
PBS	–	–	5	IM
Env_M	MR766 strain Envelope protein	Alum (500) + MPL (20)	5	IM
Env_Z	ZBRX6 strain Envelope protein	Alum (500) + MPL (20)	5	IM

Next, we analyzed the antibody subclasses to determine the detailed immune profile induced by Env_M and Env_Z. The type I cytokine IFN-γ activates IgG2 production whereas type II cytokines (e.g., IL-4) promote IgG1 expression^20,21^. When we immunized mice with Env_M, the IgG2c/IgG1 ratio was about 0.5 at both 28 and 49 dpi, while the ratio with Env_Z immunization was 0.1 (Fig. 2C,D). These results imply that Env_M and Env_Z immunization induced type II immune responses during ZIKV infection.

DENV belongs to the same flavivirus family as ZIKV and has similar amino acid sequences in E, prM, and NS1, which are targets for ZIKV vaccines^22^. A recently published study reported that DENV2 and ZIKV E protein sequences share a very similar superimposable structure with 53.9% amino acid sequence identity, and in addition, that DENV immune serum neutralizes ZIKV in vitro^23^. In this regard, we evaluated whether the immune sera of Env_M- and Env_Z-immunized mice could neutralize DENV using a plaque reduction neutralization test. The titers of neutralizing antibodies against ZIKV infection (MR766 and PRVABC59) were 1:15 ~ 20 at 49 dpi with Env_M and Env_Z, similar to the neutralizing ability against ZIKV (Table 2).

**Table 2:** Detection of neutralizing antibodies against Zika virus (ZIKV) and dengue virus (DENV-2) in immunized mice.

Groups	PRNT_50_ Titer		
	ZIKV (MR766)	ZIKV (PRVABC59)	DENV-2
PBS	0	0	0
Env_M	20	20	20
Env_Z	15	20	20

### Immunization with Env_M and Env_Z induced cellular immunity to ZIKV

**Figure 3:**
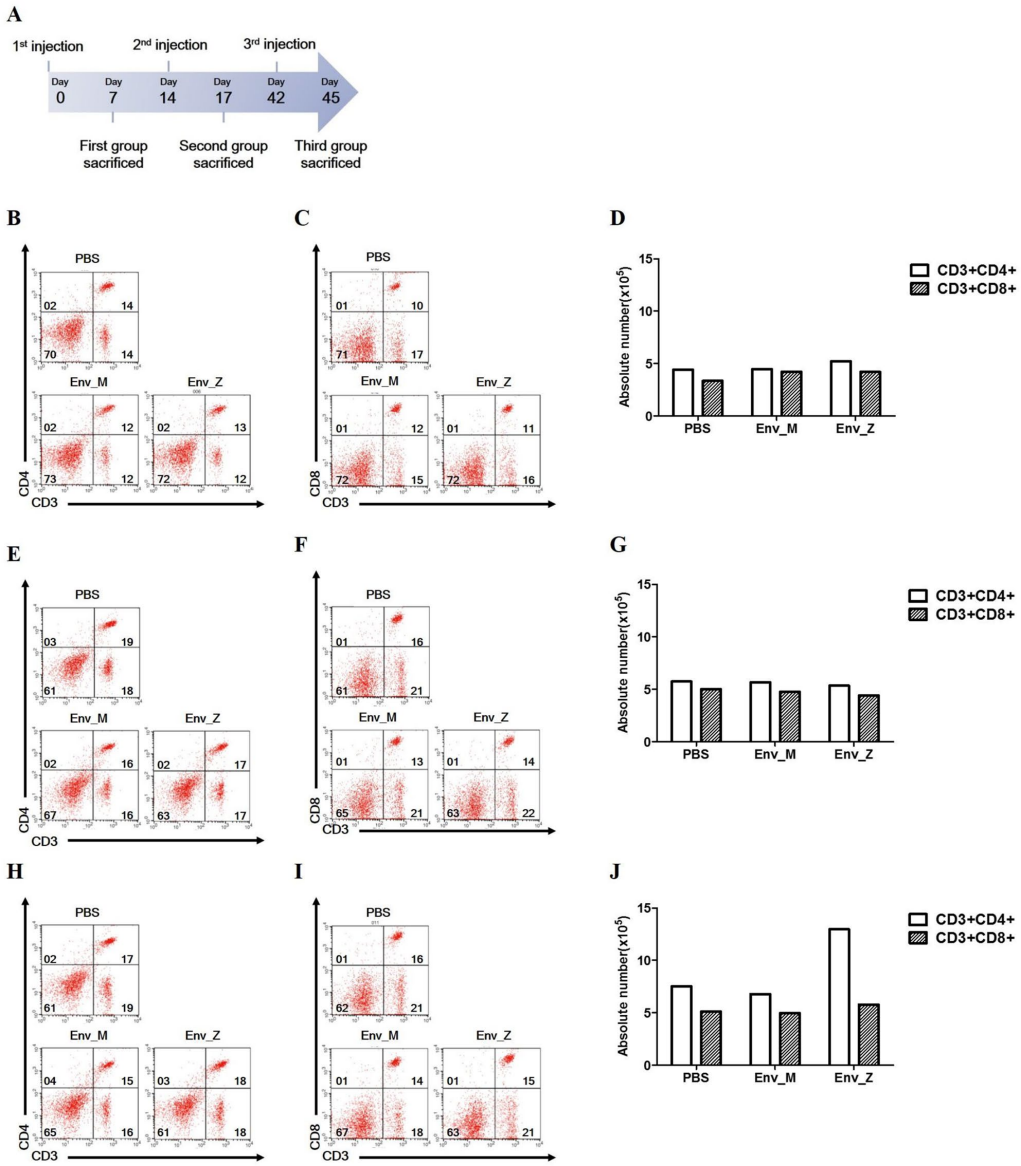
Effect of recombinant vaccination (1 ~ 3 doses) on CD4^+^ and CD8^+^ T cell population was examined. (**A**) Experimental strategies to measured cellular immune response. C57BL/6 mice (n = 5) were immunized with ZIKV Env_M, Env_Z or PBS at weeks 0, 2 and 6. Mice were euthanized 7 (Day 7; **B**–**D**, Day 17; **E**–**G**) or 3 (Day 45; **H**–**J**) days after the last immunization. Splenocytes were isolated and pooled from five mice per each group. Cells were stained for CD3, CD4, CD8 antibody and were gated CD3^+^, CD4^+^ or CD8^+^.

We assumed that the recombinant vaccination would regulate T cell activity, thus populations of CD4 + and CD8 + T cell subsets were compared (Fig. 3). Although the overall numbers of these T cells were not changed by single or double immunization, triple immunization boosted the CD4 + T cell population compared with controls.

**Figure 4:**
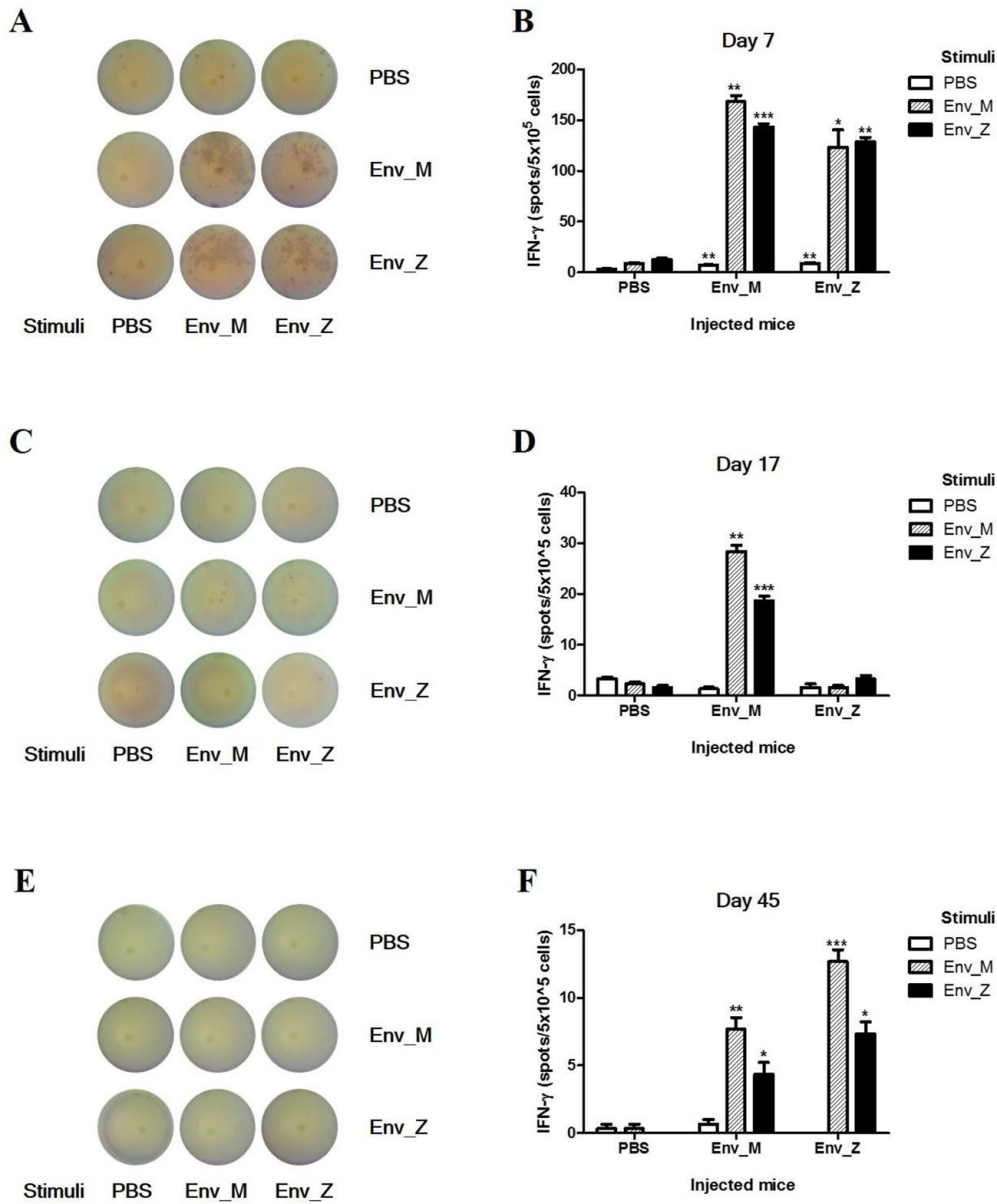
Quantification of IFN-γ secretion among Ag-specific splenocytes following recombinant vaccination was analyzed by ELISPOT. C57BL/6 mice (n = 5) were inoculated with ZIKV Env_M, Env_Z or PBS at weeks 0, 2 and 6. Mice were euthanized 7 (Day 7; **A**,**B**, Day 17; **C**,**D**) or 3 (Day 45; **E**,**F**) days after the last immunization. Splenocytes were isolated and pooled from five mice per each group. Splenocytes were stimulated with same dose of (1 μg/ml) Env_M, Env_Z or PBS. Env_M and Env_Z specific IFN-γ responses were identified by ELISPOT assay at 48 h after stimulation. (**A**,**C**,**E**) Representative ELISPOT results. (**B**,**D**,**F**) Summary of the ELISPOT results. The data were represented the mean ± SD of five mice per group. Each sample was assayed in triplicates. Statistical analysis was performed comparing immunized group with PBS injected animals and represented *p < 0.05, **p < 0.01, ***p < 0.001.

**Figure 5:**
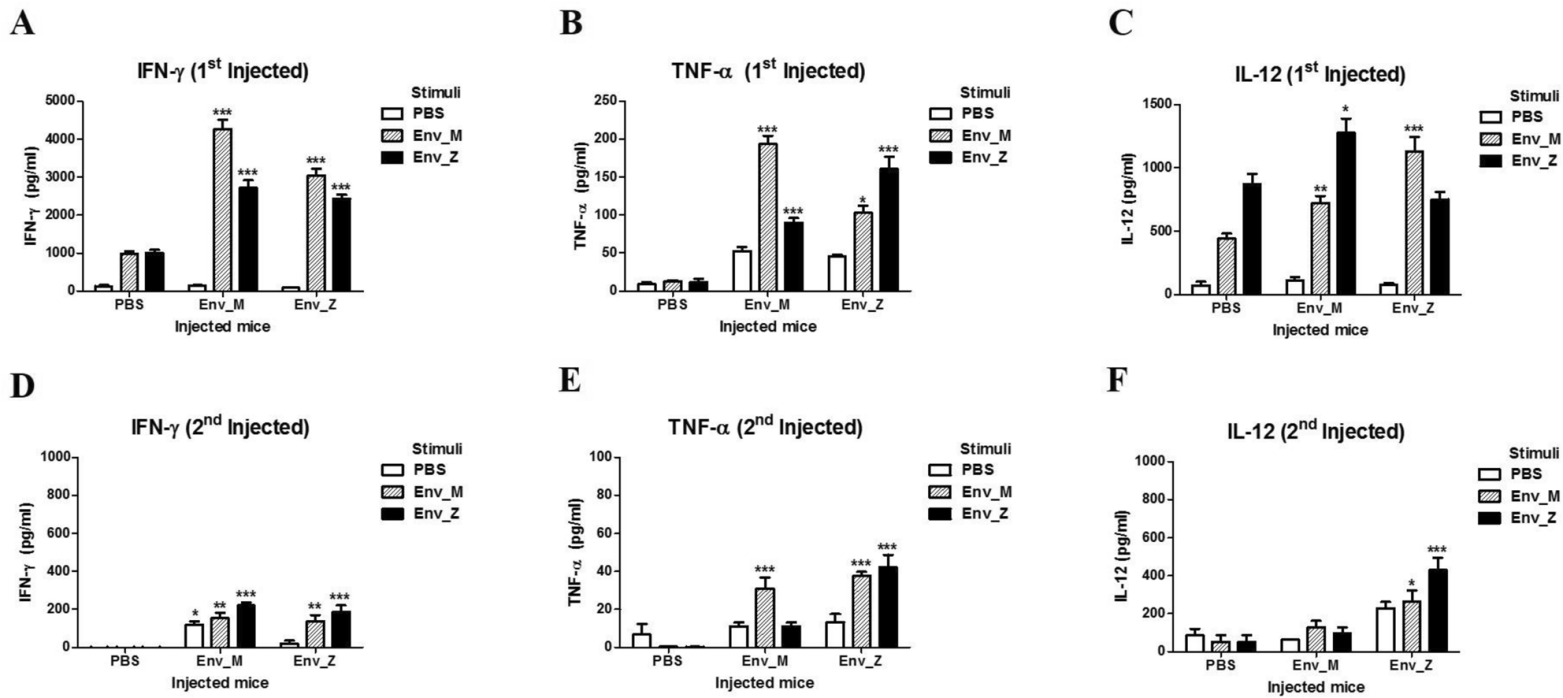
Effect of repeated Env_M, Env_Z immunization on effector cytokines production in splenocytes. C57BL/6 mice (n = 5) were immunized with ZIKV Env_M, Env_Z or PBS at weeks 0, 2. Mice were euthanized 7 (Day 7; **A**–**C**, Day 17; **D**–**F**) days after the last immunization. Splenocytes were isolated and pooled from five mice per each group. Splenocytes were stimulated with same dose of (1 μg/ml) Env_M, Env_Z or PBS. The supernatants harvested after 48 h of incubation and used to measure the concentrations of IFN-γ, TNF-α and IL-12 using ELISA. The data were represented the mean ± SD of five mice per group. Each sample was assayed in triplicates. Statistical analysis was performed comparing immunized group with PBS injected animals and represented *p < 0.05, **p < 0.01, ***p < 0.001.

Next, we examined how the T cell population mediated the cellular immunity by quantifying the IFN-γ-producing cells using ELISpot. A single immunization with Env_M and Env_Z promoted IFN-γ production from antigen-specific splenocytes. For example, Env_M injection increased the number of IFN-γ-producing cells by 19 times compared with the unimmunized group (Fig. 4A,B). By contrast, double immunizations with Env_M or Env_Z lowered the frequency of IFN-γ-producing cells by six and 38 times, respectively, compared with a single dose (Fig. 4C,D). The majority of IFN-γ-producing cells disappeared following triple immunization (Fig. 4E,F). To investigate cytokine production further, we measured circulating levels of IFN-γ, IL-12, and TNF-α after Env_M and Env_Z immunization using ELISA. A single immunization with Env_M or En v_Z induced IFN-γ levels approximately 30 times compared with nonimmunized mice, but repeated immunizations inhibited the expression of IFN-γ more than 90% (Fig. 5A,D). IL-12 and TNF-α are pro-inflammatory cytokines released from antigen-presenting cells^24,25^ and these cytokines were also promoted by a single immunization but inhibited after repeated immunizations (Fig. 5B,C,E,F).

### Immunization with ZIKV Env_M, Env_Z confers protection against ZIKV or DENV challenge in immunocompetent neonatal mice

**Figure 6:**
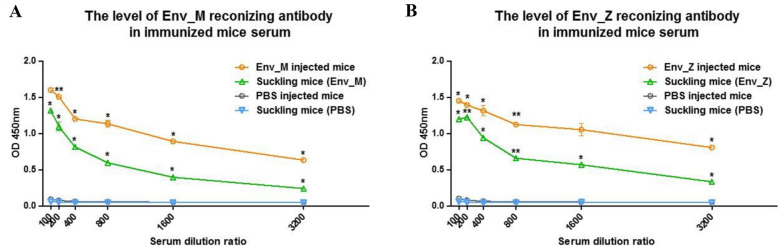
Level of vaccine specific maternal antibody in neonates’ circulation. C57BL/6 mice (n = 5) were immunized with ZIKV Env_M, Env_Z, or PBS at weeks 0, 2 and 6. Immunized mice bred as homozygous breeding pairs. To measured Env_M (**A**) or Env_Z (**B**) specific IgG antibodies, blood samples were collected on Day 2. Serum samples were twofold serially diluted from 1:100 to 1:3200 and measured for OD 450 values by using ELISA. The data were represented the mean ± SD of five mice per group. Each sample was assayed in duplicates. Statistical analysis was performed comparing immunized group with PBS injected animals and represented *p < 0.05, **p < 0.01, ***p < 0.001.

**Figure 7:**
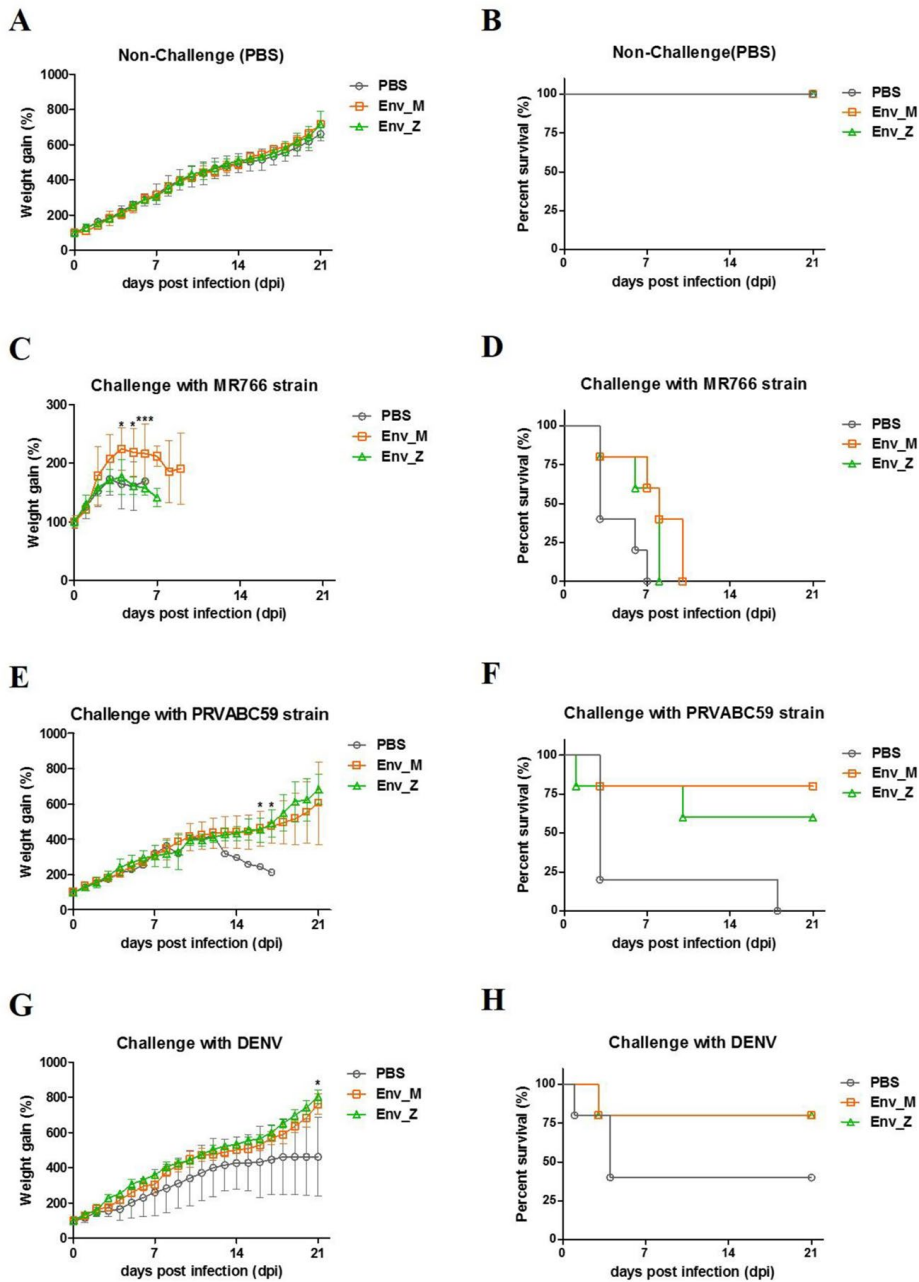
Evaluation of protective effect of recombinant vaccine against ZIKV or DENV challenge. Female C57BL/6 mice (n = 5) were administrated with ZIKV Env_M, Env_Z, or PBS at the week of 0, 2, & 6, and were bred with their homozygous males (C57BL/6). Two-day-old neonatal mice were inoculated with PBS (**A**,**B**), 10^6.8^ TCID_50_/mouse of MR766 strain (**C**,**D**), 10^6.3^ TCID_50_/mouse of PRVABC59 strain (**E**,**F**) or 10^6^ TCID_50_/mouse of DENV-2 strain (**G**,**H**). The survival rate and body weight change of neonatal mice (n = 5 per group) was monitored until 3 weeks post infection. The body weight data were represented the mean ± SD of five mice per group (**A**,**C**,**E**,**G**). The survival rate were represented the Kaplan–Meier survival curves (**B**,**D**,**F**,**H**). Statistical analysis was performed comparing immunized group with PBS injected animals and represented *p < 0.05, **p < 0.01, ***p < 0.001.

Because transmission of the virus through the placenta is fatal to the fetus and neonates, control of ZIKV during early pregnancy is critical^26^. Hence, we evaluated the protective efficacy of our vaccine in terms of induction of maternal antibodies and the survival rate of infected neonates. Before breeding, female mice were immunized with Env_M and Env_Z. Their pups had significant amounts of circulating vaccine-specific IgG within 2 days of birth (Fig. 6). Next, pups were challenged with two ZIKV strains (MR766 and PRVABC59) and we examined the viremia after 2 days of infection. After MR766 infection, neither Env_M nor Env_Z immunizations reduced circulating virus significantly and pups lost weight or did not survive (Fig. 7A–D). However, when mice were challenged with PRVABC59, Env_M vaccination successfully suppressed viral proliferation and elicited the survival rate. Of interest, ZIKV was not detected in the Env_M injected group for which the survival rate reached 80% (Tables 3 and 4, Fig. 7E,F).

**Table 3:** Assessment of viremia in neonatal mice serum.

Groups	TCID_50_/ml		
	African ZIKV strain (MR766)	Asian ZIKV strain (PRVABC59)	DENV-2
PBS	10^2.5^	10^2.3^	10^2.5^
Env_M	10^2.3^	0	10^2.5^
Env_Z	10^2.5^	10^1.7^	10^2.5^

**Table 4:** Clinical presentation in suckling mice infected with ZIKV or DENV.

Challenge	Vaccine	No. of mice	Symptom		
			Feeble (%)	Staggering march (%)	Death (%)
PBS	PBS	5	0 (0/5)	0 (0/5)	0 (0/5)
	Env_M	5	0 (0/5)	0 (0/5)	0 (0/5)
	Env_Z	5	0 (0/5)	0 (0/5)	0 (0/5)
African ZIKV strain (MR766)	PBS	5	100 (5/5)	0 (0/5)	100 (5/5)
	Env_M	5	100 (5/5)	0 (0/5)	100 (5/5)
	Env_Z	5	100 (5/5)	0 (0/5)	100 (5/5)
Asian ZIKV strain (PRVABC59)	PBS	5	100 (5/5)	100 (5/5)	100 (5/5)
	Env_M	5	40 (2/5)	0 (0/5)	40 (2/5)
	Env_Z	5	40 (2/5)	20 (1/5)	20 (1/5)
DENV-2	PBS	5	80 (4/5)	0 (0/5)	60 (3/5)
	Env_M	5	20 (1/5)	0 (0/5)	20 (1/5)
	Env_Z	5	20 (1/5)	0 (0/5)	20 (1/5)

We also assessed whether maternal immunity via Env_M and Env_Z immunizations provided cross protection to DENV (type 2) infection. In parallel with ZIKV-induced viremia, although pups from immunized adults failed to clear DENV from circulation (Table 3), their survival rate after DENV infection was significantly elicited (Fig. 7G,H, Table 4). These results suggest that the Env_M and Env_Z recombinant vaccines deliver protective immunity to the next generation.

## Discussion

ZIKV has been a public health threat for several decades, with a threatened epidemic. Developing a ZIKV vaccine is critical for both epidemic control and in preparation of its global spread^2,3^. However, no ZIKV vaccine has been approved despite several preclinical trials with purified-inactivated, live-attenuated, DNA, mRNA, and recombinant vaccines^15^. For example, ZIKV purified-inactivated virus is considered a relatively safe method for immunizing immunodeficient populations but its efficacy is low that requires repeated vaccinations^27^. A live-attenuated vaccine has the potential risk of transition to a virulent form that induces toxic inflammatory responses^28^. By contrast, a subunit vaccine is safe enough to immunize broad populations, even during pregnancy^28^. For our specific method, the baculovirus expression system is considered a reliable approach that is only virulent to certain insect species. In addition, highly purified antigen produced by the baculovirus system induces potent immune responses to specific protein epitopes. A recombinant E protein from an insect cell line acted as a booster inducing protective immunity to West Nile virus infection^16,29–31^.

Because we recognized the ZIKV E protein as the main target of neutralizing antibodies and the source of the distinctive pathogenicity of Asian (PRVABC59) and African (MR766) strains^32^, we developed a recombinant vaccine strategy involving viral envelope protein glycosylation using the baculovirus expression system. Correct glycosylation is essential for the active interaction between antigen and host-cell receptors that induces protective immunity against ZIKV infection. To evaluate the adaptive immunity induced by vaccination, we first examined the humoral immune response and detected antigen-specific antibodies. Plasma cells secrete ZIKV-specific immunoglobulins that recognize epitopes from the E protein dimer–dimer interface. The significance of this humoral immunity was confirmed in mouse models that showed vaccination protected against vertical transmission and high mortality in a B cell-deficient host. Moreover, adoptive transfer of immune IgG protected against ZIKV infection, which confirms the efficacy of the neutralizing antibody^5,6,30^. Therefore, authorized vaccines for other flaviviruses and current ZIKV vaccines in development focus on the induction of adequate neutralizing antibodies^9^. Consistent with this, we observed that both Env_M and Env_Z immunizations established potent humoral immunity that induced ZIKV E protein-specific IgG and neutralizing antibodies. Authorized ZIKV vaccine is yet to be developed but the effective neutralization titers of approved flavivirus vaccines are 1:10^28,33^. Our Env M and Env Z vaccines showed improved titer (1:20) against ZIKV and DENV challenge which supports the efficacy of our vaccine strategy.

To provide optimal immunity during viral infection, both humoral and cellular responses should be functional, and successful vaccination results in consistent residual antibody, antigen-specific antibody, and T cell induction. CD4 + T cells and their cytokines promote the germinal-center reaction and production of ZIKV-specific antibodies. During ZIKV infection, humans, nonhuman primates, and mice generated virus-specific CD4 + and CD8 + T cells. When those T cells were adoptively transferred, the viral titer was dramatically reduced^28,34–36^. Effector cytokines such as IFN-γ, IL-12, and TNF-α are essential for viral clearance. The production of IFN-γ during innate immune responses mediates anti-ZIKV activity and the natural killer (NK) cell response^34,37^. IL-12 is produced by antigen-presenting cells and mediates induction of type 1 T cell differentiation, which leads to an antiviral response. This cytokine also mediates IFN-γ and TNF-α generation by stimulating other T cells and NK cells^38,39^. TNF-α interferes with viral entry and blocks virus replication. Previous studies observed a protective role of TNF-α during other flavivirus infections including West Nile virus and DENV^40,41^. Our vaccine generated these cytokines both in splenocytes and systemically, confirming that protective immunity was mediated by cellular responses. The ELISPOT result showed that IFN-g secretion is well maintained by Env_Z up to 45 dpi. And, Ag-specific antibody had been detected till 49 dpi. Although it is hard to conclude that Env_M induced Th1 dominant response while Env_Z is not, Env_M might have induced more balanced response than Env_Z.

Notwithstanding this boosted immunity, we observed that repeated immunization limited the expression of effector cytokines. It is possible that chronic infection induces most central memory T cells transition into effector memory T cells. Those are terminally differentiated and produce effector cytokines but short lived which is frequently seen when T cell is in anergic state^42–45^. Unexpectedly, triple immunization generated a CD4 + T cell rebound, which might be associated with the induction of regulatory T cells or T helper 2 cell expansion^46^.

An adjuvant is a useful tool to maximize vaccine efficacy and alum and MPLA were used for this study. Whereas MPLA is a toll-like receptor-4 agonist that supports type I immunity, alum is known to promote type 2 responses. The lower IgG2c/IgG1 ratio induced by repeated immunizations suggests that the action of alum is dominant and supports the induction of type 2 immunity, while alum and MPLA did not affect T cell activity including cytokine expression and germinal center formation (S.Figs. 2 and 3). However, the precise mechanisms by which the effect of adjuvant is modified by repeated vaccination must be further elucidated to better inform the vaccine strategy against ZIKV infection.

ZIKV is highly pathogenic because its transplacental transmission is lethal to the fetus. During early pregnancy, ZIKV invades the fetal brain and induces neurodestructive disorders such as microcephaly^47–50^. In addition to the primary humoral immunity induced by Env_M and Env_Z vaccination, the vaccine delivered protection to progeny. Neural signs and body weight loss were obvious in neonatal mice in the control group but pups from Env_M- and Env_Z-immunized animals showed milder clinical symptoms. This finding demonstrates that the recombinant vaccine-induced ZIKV-specific maternal antibodies were delivered efficiently to progeny. E proteins of various flaviviruses share analogous amino acid sequences and structural homology that promotes unwanted antibody cross reactivity and antibody-dependent enhancement (ADE)^51,52^. Viremia in neonate from vaccinated female was controlled against DENV challenge and promoted survival of pup. Therefore, we are reasoning that the vaccination have provided cross protection against viral infection, alleviating the risk of ADE.

The ZIKV outbreak peaked in South America in 2016 affecting millions of people and declined after 2017. From the time of this epidemic, studies have attempted to develop safe and efficient vaccines, but face obstacles in clinical studies^28^. In addition, there remains a risk of a ZIKV pandemic because of climate change-mediated expansion of the habitat of the viral vector, ineffective mosquito control, and genetic modification of ZIKV^53^. Therefore, continuous efforts are needed to develop an advanced vaccine strategy.

In summary, we developed a ZIKV E protein recombinant vaccine that generated both humoral and cellular responses that provided protective immunity in immunocompetent animals. This study highlights the importance of E protein as a target of a vaccine strategy against ZIKV infection.

## Methods

### Cells and viruses

Vero cells (KCLB, Seoul, Korea) were grown in Minimum Essential Medium (MEM)*-*α supplemented with 10% fetal bovine serum (FBS; Gibco, Grand Island, NY, USA), and Vero 76 cells (ATCC, Manassas, VA, USA) were grown in Dulbecco’s Minimal Essential Medium (DMEM; Gibco, Carlsbad, CA, USA) supplemented with 10% FBS at 37 °C in 5% CO_2_ until they formed monolayers. *Spodoptera frugiperda 9* (Sf9) and ExpiSf9 insect cells were cultured in Sf-900III SFM medium (Gibco, Grand Island, NY) at 27 °C in an orbital shaker. The ZIKV strains PRVABC59 (BEI Resources No. NR-50240) and MR766 (BEI Resources No. NR-50065) were obtained from BEI Resources. The ZIKV PRVABC59 strain was propagated in Vero cells and the ZIKV MR766 strain in Vero 76 cells at a multiplicity of infection of 0.01. ZIKV stocks were titrated by plaque assay using both cell lines and stored at –80 °C.

### Plasmid construction and protein expression

The recombinant baculoviruses were commercially constructed (GenScript, Piscataway, NJ, USA) by Bac-to-Bac expression system. Briefly, ZIKV (MR766 and ZBRX6 strain) E protein DNA sequences were synthesized and then subcloned into the pFastBac1 vector under the polyhedrin promoter (Pph), generating pFB-Env_Z and pFB-Env_M. The recombinant pFB-Env_Z and pFB-Env_M plasmids were transformed into DH10Bac competent cells. Sf9 cells were grown in Sf-900 II SFM Expression Medium (Gibco, Grand Island, NY) and were maintained in Erlenmeyer flasks at 27 °C in an orbital shaker. One day before transfection, the Sf9 cells were seeded at an appropriate density in 6-well plates (Corning-Costar, USA). On the day of transfection, Bacmid DNA and transfection reagent (Promega, Madison, USA) were mixed at an optimal ratio and then added into the 6-well plate. The transfected Sf9 cells were incubated in Sf-900 II SFM for 5–7 days at 27 °C before harvest. The supernatant was collected by centrifugation and designated as P1 viral stock. P2 virus was amplified for later infection: a 1 L Sf9 cell culture was infected with P2 virus, then cells were incubated in Sf-900II SFM (1 ×) for 3 days at 27 °C before harvest. Cell pellets were harvested and lysed using an appropriate cell lysis buffer. After centrifugation, the cell pellets were dissolved using urea. Recombinant Env_Z and Env_M proteins were obtained by one-step purification using Ni–NTA affinity chromatography. Higher purity fractions were pooled and sterilized through a 0.22 μm filter. The purified protein were analyzed using sodium dodecyl sulfate–polyacrylamide gel electrophoresis (SDS-PAGE) and Western blot analysis for molecular weight and purity measurements. The primary antibody for Western blot was mouse-anti-His mAb (GenScript, Piscataway, NJ, USA). The protein concentration was determined using a Bradford protein assay with bovine serum albumin (BSA) standard curve.

### Mouse immunization

C57BL/6 (female, 8-weeks-old) mice were purchased from Orient Bio Inc (Gyeonggi-do, Korea). Mice were housed in the Animal Laboratory Center of Kangwon National University under a 12-h light–dark cycle and given free access to food and water. Three groups of 8-week-old female C57BL/6 mice (n = 5 in each group) were vaccinated with 10 μg recombinant ZIKV E protein (Env_Z, Env_M) or phosphate buffered saline (PBS) (control). The vaccine was mixed with 500 μg of aluminum hydroxide gel (Invivogen, San Diego, CA, USA) and 20 μg of monophosphoryl Lipid A (MPLA; Sigma‐Aldrich, St. Louis, MO, USA). The vaccine–adjuvant mixture was administered via the intramuscular route at days 0, 14, and 42. For cellular immune response studies, splenocytes were collected at each vaccination point (7, 17, 45 days post initial immunization [dpi]). Mouse sera were collected at 0, 14, and 49 dpi and immunized mice were mated with homozygous males. One-day-old neonates were inoculated subcutaneously with each strain of ZIKV (MR766, PRVABC59) or PBS as a negative control. Mice were inoculated with 10^6.8^ tissue-culture-infected doses (TCID)_50_/mouse of MR766 strain or 10^6.3^ TCID_50_/mouse of PRVABC59 ZIKV strain and were euthanized with CO_2_ at 21 dpi. Serum was collected at 0 and 2 dpi. This work was carried out in compliance with the ARRIVE guidelines and approved by the Institutional Animal Care and Use Committee of Kangwon National University (No. KW-190131-1, KW-190515-2). All methods were carried out in accordance with relevant guidelines and regulations.

### Enzyme-linked immunosorbent assay (ELISA)

The sera were tested for ZIKV E protein-specific antibodies using commercially available ELISA kits (Alpha Diagnostic, USA) according to the manufacturer’s instructions. Briefly, 100 μl aliquots of diluted serum (1:50,000) were added per well. Env_M- or Env_Z-specific antibodies (IgG, IgG1, and IgG2c) in sera were determined by indirect ELISA. Microplates (Nunc-Immuno Plates; Thermo Scientific, UK) were coated with Env_M (1 μg/ml) at 4 °C overnight. Each well was washed with 0.05% Tween 20 in PBS (PBST) and then with 1% BSA in PBS and incubated at 37 °C for 2 h. The plates were washed and then diluted serum was incubated at 37 °C for 2 h. Plates were washed and incubated with horseradish peroxidase-conjugated goat anti-mouse IgG heavy and light chain antibody (1:10,000; Bethyl Laboratories, TX, USA), goat anti-mouse IgG1 (1:10,000; Bethyl Laboratories), or goat anti-mouse IgG2c (1:8000; Southern Biotech, USA) at 37 °C for 1 h and washed with PBST. Color development was performed using tetramethylbenzidine substrate (Surmodics, USA) and stopped with 2 N H_2_SO_4_. The optical density of the plates was read at 450 nm in an ELISA plate reader (BioTek, Winooski, NT, USA).

### Plaque reduction neutralization test

Vero or Vero 76 cells were seeded in 24-well plates at 1 × 10^5^ cells/well and cultured overnight in DMEM medium containing 10% FBS. Serum samples were heat inactivated at 56 °C for 30 min and serially diluted in DMEM. ZIKV and DENV were was diluted to 2 × 10^2^ plaque-forming units (PFU)/ml in DMEM, mixed with an equal volume of diluted serum and incubated at 37 °C for 30 min. Then, Vero or Vero 76 cells were incubated with the serum mixture at 37 °C for 2 h. Cells were then washed and cultured at 37 °C for 5 or 14 days in DMEM containing 1% sea plaque agar or 1.4% methyl cellulose. Cells were fixed in 4% paraformaldehyde and stained with 0.1% crystal violet dye. The percentage inhibition of virus infectivity was calculated by counting the number of plaques in immune sera.

### Measurement of cytokine and T lymphocyte subtypes

The interferon (IFN)-γ secreting splenocytes were measured at 7, 17, and 45 days after the first immunization using mouse IFN-γ enzyme-linked immune absorbent spot (ELISpot) assay (BD Life Sciences, USA). Briefly, 5 × 10^5^ splenocytes/well were stimulated with Env_M (1 μg/ml) or Env_Z (1 μg/ml). Spots were counted under a dissecting microscope (Olympus, model no. SZH-ILLB).

For cytokine analysis, 5 × 10^5^ splenocytes/well were stimulated with Env_M (1 μg/ml) or Env_Z (1 μg/ml). The media from splenocyte cultures were collected after 48 h incubation. The levels of cytokines were determined using mouse tumor necrosis factor (TNF)-α ELISA MAX™ standard set, mouse IFN-γ ELISA MAX™ standard set, and mouse interleukin (IL)-12 ELISA MAX™ standard set (BioLegend, USA). Each assay was performed in triplicate.

To analyze T lymphocyte subtypes, splenocytes were stained with the following antibodies at a dilution of 1:200 with 1% BSA in PBS at 4 °C for 1 h: anti-mouse CD3 (fluorescein isothiocyanate, BD Biosciences), and anti-mouse CD4 (phycoerythrin [PE], BD Biosciences) or anti-mouse CD8 (PE, BD Biosciences). Cell phenotype was analyzed using a FACSCalibur flow cytometer (Becton Dickinson).

### Tissue culture infective dose_50_ (TCID_50_)

Vero or Vero 76 cells were seeded in 96-well plates at 2 × 10^4^ cells/well and cultured overnight in DMEM medium containing 10% FBS. Serum samples were heat inactivated at 56 °C for 30 min and serially diluted (10^1^–10^3^) in PBS. The serum samples were used to infect Vero or Vero 76 cells at 37 °C for 1 h. Cells were then washed and cultured at 37 °C for 14 days in complete DMEM. TCID_50_ was calculated using the end point method, and virus infectivity was determined using the Karber method.

### Statistical analysis

Statistical analysis was performed using GraphPad Prism (v. 5.0; GraphPad Software, La Jolla, CA, USA) using one-way analysis of variance with Tukey’s multiple comparisons test to compare groups. *P* values < 0.05 were considered significant.

## Supplementary Information

Supplementary Information.
