# Supplementary material for: Zika virus baculovirus-expressed envelope protein elicited humoral and cellular immunity in immunocompetent mice
Source: Sci Rep. 2022 Jan 13;12:660. doi: 10.1038/s41598-021-04713-7 (PMC8758750; doi:10.1038/s41598-021-04713-7)

**Supplementary Data**

**Supplementary Table 1.** Detection of neutralizing antibodies against ZIKV in immunized mice.

| Group | Injection Ag | Adjuvant | PRNT_50_ Titer | |
| --- | --- | --- | --- | --- |
|  |  |  | MR766 | PRVABC59 |
| 1 | - | - | <10 | <10 |
| 2 | - | Alum + MPLA | <10 | <10 |


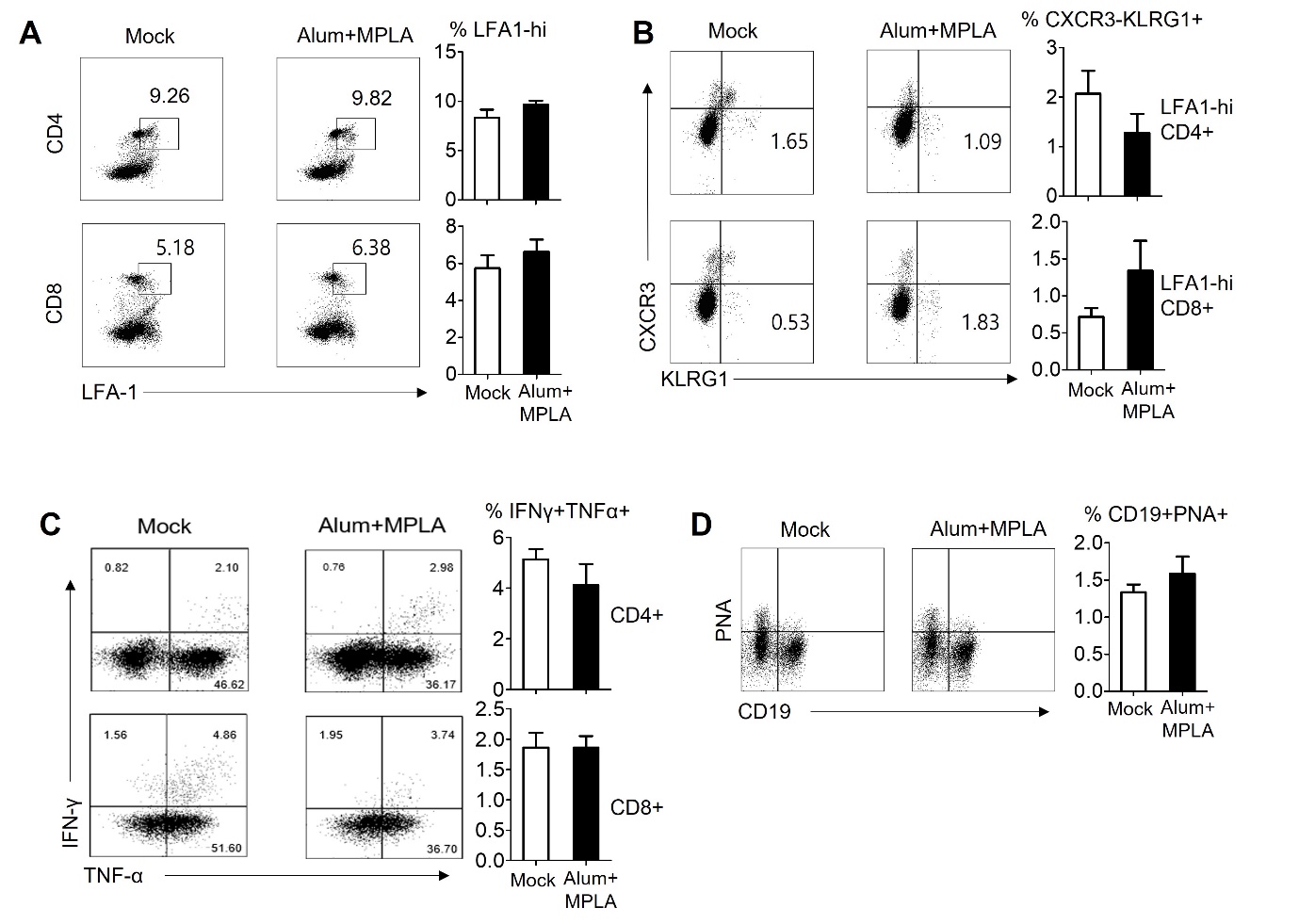
**Supplementary Figure 1.** Effect of adjuvant (alum+MPLA) on T cell and B cell responses in splenocytes. (A) T cell activation was evaluated with LFA-1 surface marker. (B) Level of effector memory T cell (CXCR3-KLRG1+) differentiation by adjuvant inoculation. (C) Effector cytokine production by adjuvant inoculation. (D) Germinal center formation was analyzed by CD19 and PNA staining.

**Supplementary Figure 2.** Production of effector cytokines by adjuvant inoculation.


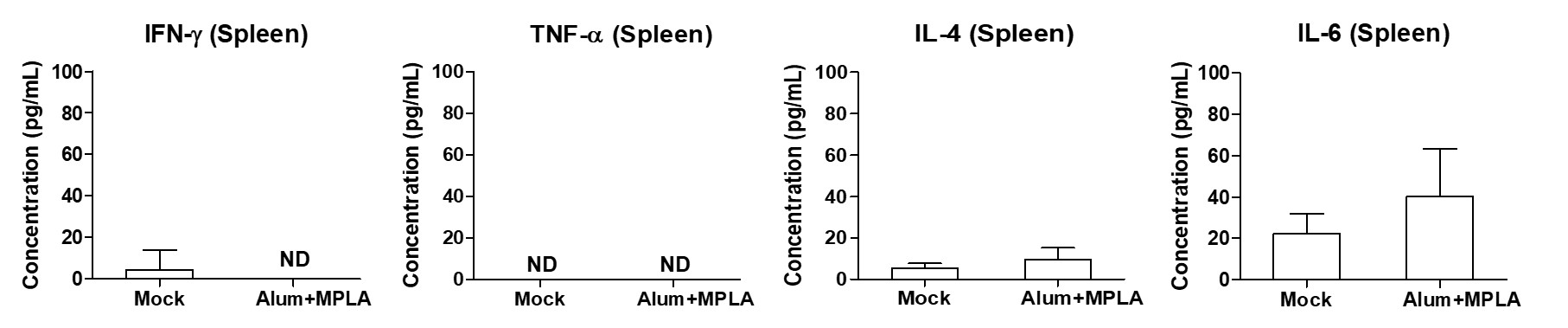

Supplement: Supplementary file 1 — Supplementary Information. [file 41598_2021_4713_MOESM1_ESM.docx]
